# The Great International Congress on Tuberculosis, St. Louis, October 3, 4, and 5

**Published:** 1904-09

**Authors:** 


### The Great International Congress on Tuberculosis, St. Louis, October 3, 4, and 5.

This Congress is not intended to discuss medical questions *per se.* Treatment outside of the relation of State legislation as to sanatoria will not be before the Congress.

The contentions of the Bacteriologists and the Pathologists; of Koch and Behring, and Von Schroen, Marmorel and others, are interesting, but they sink in significance when compared with the problems that now arrest and command the attention of the ablest thinkers of the world, in all professions throughout all lands. It is not possible to read before the Congress all the contributions likely to be made from the great number of countries of the Western Hemisphere, who have been invited by the Government of the United States to send representatives to this Congress, so many of whom have accepted.

It is most important that not only the general public, but medical men especially should recognize and emphasize the fact that this Congress was organized and intended as a Medico-Legal and popular effort to extend the public sanitation of tuberculosis on lines indicated by medical advance, but not yet generally carried out. It is the mission of this Congress to popularize and spread needed sanitation on these lines. The fact is that the public needs, now ask for means, methods, and proper organization of all classes, and that it should not be composed of or limited to medical men only. These are questions which, in addition to preventive legislation, this Congress should explain and develop, and especially should it arrange to co-operate with and help every organized movement now commenced or to be started by State and municipal bodies, which look to the education of the masses of our people, in every well directed effort to miminize the terrible devastation of this scourge of mankind.—*Medico-Legal Journal.*

